# T Regulatory Cell Subsets Do Not Restore for One Year After Acute COVID-19

**DOI:** 10.3390/ijms252111759

**Published:** 2024-11-01

**Authors:** Arthur Aquino, Ekaterina Zaikova, Olga Kalinina, Tatiana L. Karonova, Artem Rubinstein, Arina A. Mikhaylova, Igor Kudryavtsev, Alexey S. Golovkin

**Affiliations:** Almazov National Medical Research Centre, 197341 St. Petersburg, Russia; akino97@bk.ru (A.A.); arrubin6@mail.ru (A.R.); igorek1981@yandex.ru (I.K.)

**Keywords:** COVID-19, T regulatory cells, convalescent period, purinergic signaling, multicolor flow cytometry

## Abstract

COVID-19, caused by SARS-CoV-2, triggers a complex immune response, with T regulatory cells (Tregs) playing a crucial role in maintaining immune homeostasis and preventing excessive inflammation. The current study investigates the function of T regulatory cells during COVID-19 infection and the subsequent recovery period, emphasizing their impact on immune regulation and inflammation control. We conducted a comprehensive analysis of Treg subpopulations in peripheral blood samples from COVID-19 patients at different stages: acute infection, early convalescence, and long-term recovery. Flow cytometry was employed to quantify Tregs including “naïve”, central memory (CM), effector memory (EM), and terminally differentiated CD45RA^+^ effector cells (TEMRA). Additionally, the functional state of the Tregs was assessed by the expression of purinergic signaling molecules (CD39, CD73). Cytokine profiles were assessed through multiplex analysis. Our findings indicate a significant decrease in the number of Tregs during the acute phase of COVID-19, which correlates with heightened inflammatory markers and increased disease severity. Specifically, we found a decrease in the relative numbers of “naïve” and an increase in EM Tregs, as well as a decrease in the absolute numbers of “naïve” and CM Tregs. During the early convalescent period, the absolute counts of all Treg populations tended to increase, accompanied by a reduction in pro-inflammatory cytokines. Despite this, one year after recovery, the decreased subpopulations of regulatory T cells had not yet reached the levels observed in healthy donors. Finally, we observed the re-establishment of CD39 expression in all Treg subsets; however, there was no change in CD73 expression among Tregs. Understanding these immunological changes across different T regulatory subsets and adenosine signaling pathways offers important insights into the disease’s pathogenesis and provides a broader view of immune system dynamics during recovery.

## 1. Introduction

The COVID-19 pandemic caused by severe acute respiratory syndrome coronavirus 2 (SARS-CoV-2) resulted in significant morbidity and mortality worldwide [1]. The immune response to SARS-CoV-2 infection plays a crucial role in disease outcomes, ranging from asymptomatic or mild cases to multiorgan failure. High mortality and severe complications associated with COVID-19 are linked to the development of a dysregulated systemic inflammatory response characterized by a “cytokine storm” [2]. The cytokine storm can lead to the manifestation of acute respiratory distress syndrome (ARDS) and multiorgan failure, affecting organs such as the kidneys, liver, and heart [3]. Furthermore, recent data revealed that main innate and adaptive cell subsets were altered during acute SARS-CoV-2 infection [4], as well as the fact that immune cells dysregulations persisted in long COVID-19 patients even years after infection [5,6]. In this context, Tregs appear to be crucial players in the regulation and suppression of the excessive inflammatory reactions observed during SARS-CoV-2 infection and long COVID [7]. Consequently, investigation of the functional role of Tregs in the context of SARS-CoV-2 is of great importance, not only for enhancing our understanding of disease progression but also for identifying potential therapeutic targets. Some investigators have even applied Tregs therapy on COVID-19 patients and achieved successful results [8].

Tan et al. have found an increase in the absolute count of Tregs (CD3^+^CD4^+^CD25^+^CD127^low^) in patients with mild disease compared to the control group, whereas in severe cases, the increase was observed only in the relative count of the cells [9]. The expression of CD25 was increased and the expression of CD127 was decreased in the Tregs of patients with severe COVID-19 [9]. The remaining expression levels partially returned to those of healthy donors after recovery [10]. Other studies showed low levels of CD25^+^Foxp3^+^CD127^−^ Tregs in all recovering patients compared to healthy individuals [11].

Wang et al. noted a tendency for Tregs growth during the transition from mild to severe states in patients, followed by a decline during the transition from severe to critical condition [12]. They also observed that the expression levels of CXCR3, CD28, and TGF-β on Tregs were higher in patients with COVID-19 compared to healthy controls [12]. The increase in CXCR3 expression correlated with disease severity [13] and suggested the migration of these cells to the site of inflammation. Furthermore, these cells in patients with coronavirus infection exhibited a more suppressive phenotype with increased expression of Ki67, CTLA-4, GITR, and ICOS as the disease progressed [13].

Some researchers identified a reduced number of peripheral blood Tregs in patients with COVID-19 [14], both in severe and moderate cases [10]. The expression of FoxP3 also was lower than in healthy donors. Next, Th17/Tregs and RORyt/FoxP3 ratios were increased in patients with coronavirus infection [14]. Moreover, Kalfaoglu et al. showed a decrease in the level of FOXP3^+^IL2RA^+^CD4^+^ T-cells in severe patients compared to those with moderate severity [15]. On the contrary, Samaan et al. observed a sharp increase in FoxP3 expression in Tregs in patients with severe COVID-19 [16]. According to their data, the expression of such activation markers as KLRG1 and PD1 on the surface of Tregs also increased. In the severe patient group, there was an increase in T-bet^+^ T-regs, indicating protective immune responses to the pro-inflammatory environment created by Th1 during coronavirus infection. In some FoxP3^+^ Tregs of severe patients, the expression of IL-32 was increased [17]. Therefore, it can be speculated that Tregs in this infection may not only transition to an effector form but also exacerbate inflammatory reactions by releasing this cytokine.

It was observed that patients with a low number of Tregs had stronger cytotoxic follicular helper cells and cytotoxic T helper cells response, while patients with a higher level had a weak response [18]. However, other researchers did not find significant differences between FOXP3^+^ Tregs in COVID-19 patients and healthy donors [19], or they found such differences only in patients with a critical condition [20].

Finally, a limited number of studies depict the dynamic changes in levels of Tregs in the convalescent period. For instance, one study reveals an increase in the relative count of Tregs (CD4^+^CD25^+^CD127^low^) in patients with long COVID compared to those recovered from illness [21]. Conversely, another study demonstrated a decrease in Tregs in nine patients with persistent symptoms [22].

This study aims to evaluate the dynamics of Treg cell subsets and the expression patterns of ectonucleotidases (CD39 and CD73) during acute COVID-19 and the convalescent period, to better understand the pathogenesis of the disease and its long-term immune-mediated complications.

## 2. Results

### 2.1. Alterations of T Lymphocytes and Their Populations (CTL, Th, and Tregs) in Acute COVID-19

The acute phase of COVID-19 was defined as the period during which patients were hospitalized due to active viral infection, characterized by clinical symptoms such as fever, respiratory distress, and lung involvement. The severity of COVID-19 was determined based on the extent of lung involvement, as assessed by computed tomography (CT) scans, and C-reactive protein (CRP) levels. A moderate course was defined as <50% lung involvement on CT scans and CRP levels < 110 mg/L, while a severe course was defined as ≥50% lung involvement or CRP levels ≥ 110 mg/L.

**Figure 1:**
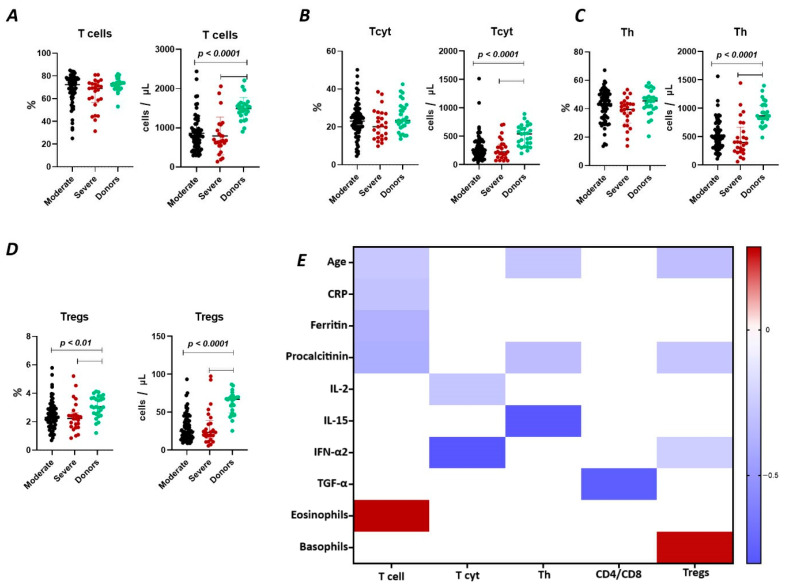
T cell subpopulations in acute COVID-19 and in healthy donors. (**A**). Relative count and absolute number of T cells. (**B**). Relative count and absolute number of cytotoxic T cells (CD3^+^CD8^+^). (**C**). Relative count and absolute number of T helper cells (CD3^+^CD4^+^). (**D**). Relative count and absolute number of T regulatory cells (CD3^+^CD4^+^CD25^bright^). (**E**). Heat map of correlations between T cell subsects and some conventional clinical and laboratory markers in patients with COVID-19.

The results show (Figure 1A) that the relative content of CD3^+^ lymphocytes did not differ between the two groups of COVID-19 patients (72.38% (64.51; 77.28) and 68.99% (58.67; 73.00) in moderate and severe cases, respectively) and compared to the donor group (73.18% (69.16; 74.06)). However, the absolute count of CD3^+^ lymphocytes in patients with moderate (782 cells/μL (527; 988) and severe infection (675 cells/μL (547; 934)) was significantly reduced (*p* < 0.0001) compared to the conditionally healthy volunteers (1495 cells/μL (1353; 1603)).

We observed a negative correlation (Figure 1) between the relative count of CD3^+^ lymphocytes and levels of C-reactive protein (r = −0.30058), ferritin (r = −0.38467), procalcitonin (r = −0.39720), and age (r = −0.27173), as well as a positive correlation with the levels of eosinophils (r = 0.28536).

It was determined (Figure 1B) that there were no significant differences in the relative count of CD8^+^ T lymphocytes in patients with moderate (23.07% (18.66; 27.20)), severe (20.75% (14.32; 27.78)) COVID-19 patients, and healthy donors (23.42% (19.55; 29.89)). However, the absolute count of CD8^+^ lymphocytes was significantly reduced in both groups of patients (248 cells/μL (157; 404) and 225 cells/μL (133; 359) in moderate and severe, respectively) compared to the donor’s group (541 cells/μL (330; 661)). Moreover, the inverse relationship was found between the absolute count of CD8^+^ T lymphocytes and levels of IL 2 (r = −0.28260) and IFN-α2 (r = −0.80244) (Figure 1).

There were no significant differences in the relative count of CD4^+^ T lymphocytes between patients with moderate (43.60% (34.37; 50.37) and severe 39.50% (31.73; 43.71)) COVID-19 patients and healthy donor’s group (45.47% (40.65; 48.62)) (Figure 1C). However, the absolute count of CD4^+^ lymphocytes was significantly decreased in both groups of patients with moderate (498 cells/μL (329; 630) and severe 400 cells/μL (248; 589) infection and compared to the donor’s group (868 cells/μL (811; 1055)). We also observed a negative correlation (Figure 1) between the levels of CD4^+^ lymphocytes and procalcitonin (r = −0.32450), IL-15 (r = −0.79394), and age (r = −0.28260).

Relative counts of Tregs were decreased in both groups of patients (2.31% (1.72; 2.93) and 2.22% (1.66; 2.48) in moderate and severe, respectively) compared to healthy donors (3.04% (2.55; 3.68) (Figure 1D). The absolute content of Treg lymphocytes was also significantly decreased in both moderate (25 cells/μL (16; 39) and severe 23 cells/μL (18; 35)) groups compared to the healthy donors (67 cells/μL (49; 70)). Furthermore, a negative correlation (Figure 1) was found between the relative count of CD3^+^CD4^+^CD25^bright^ lymphocytes and levels of procalcitonin (r = −0.27975) and age (r = −0.31491), whereas Tregs level positively correlated with basophils count (r = 0.27030).

### 2.2. Alterations in Regulatory T Lymphocyte Subpopulations in Acute COVID-19

**Figure 2:**
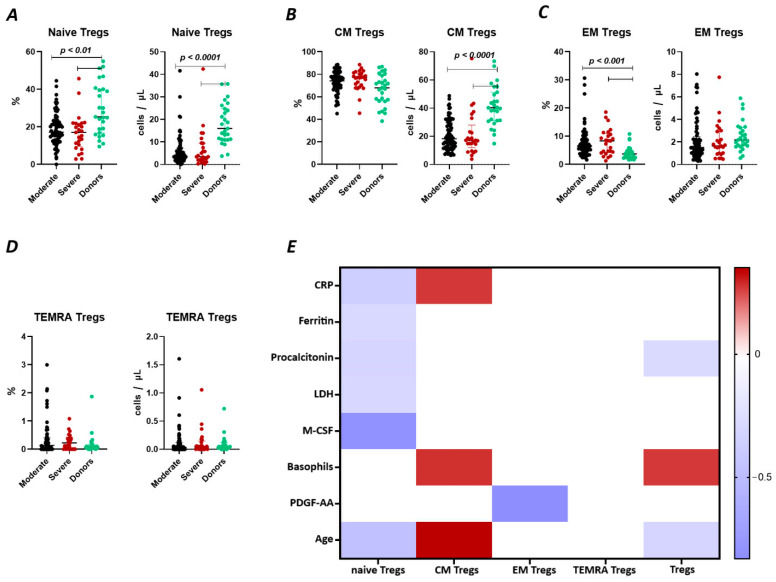
T Regulatory cell subsets in acute COVID-19 and in healthy donors. (**A**). Relative count and absolute number of naïve regulatory T cells (Naïve Tregs). (**B**). Relative count and absolute number of central memory regulatory T cells (CM Tregs). (**C**). Relative count and absolute number of effector memory regulatory T cells (EM Tregs). (**D**). Relative count and absolute number of terminally differentiated CD45RA^+^ effector regulatory T cells (TEMRA Tregs). (**E**). Heat map of correlations between regulatory T cell subsects and some conventional clinical and laboratory markers in patients with COVID-19.

We observed a significant decrease (Figure 2A) in the relative count of naive Tregs between both groups of patients with COVID-19 (17.18% (13.01; 23.16) and 17.01% (10.51; 21.84) moderate and severe, respectively) and healthy donors group (25.3% (16.75; 40.06)). We also observed a significant decrease in the absolute count of the cells. Furthermore, significant correlations (Figure 2) were found between the levels of naïve Tregs and C-reactive protein (r = −0.37310), ferritin (r = −0.29294), lactate dehydrogenase (LDH) (r = −0.32017), age (r = −0.36147), and M-CSF (r = −0.81459).

There were no significant differences (Figure 2B) in the relative count of CM Tregs, whereas absolute counts in patients with moderate (18 cells/μL (12; 29) and severe 17 cells/μL (15; 23)) infection were decreased compared to those in healthy donors (40 cells/μL (31; 51)). Moreover, we observed the correlations between the relative count of CM Tregs and levels of CRP (r = 0.27143), and age (r = 0.35186).

As opposed to naïve Tregs, the relative count of EM Tregs in patients with moderate 6.47% (4.41; 9.26) and severe 8.35% (4.43; 10.66) infection was increased compared to healthy donors (3.71% (2.48; 4.86), whereas the absolute levels of cells in the patients were equal to those in the healthy donors (Figure 2C). We observed a correlation between the relative count of EM Tregs and levels of platelet-derived growth factor-AA (PDGF-AA) (r = −0.83030) (Figure 2).

There were no significant differences in relative nor in absolute counts of TEMRA Tregs in both groups of patients and healthy donors (Figure 2D). Moreover, the levels did not correlate with any of the studied clinical or laboratory parameters (Figure 2E).

### 2.3. Alterations in the Purinergic Signaling

#### 2.3.1. Characteristics of CD39 Expression on Regulatory T Lymphocytes in Acute COVID-19

**Figure 3:**
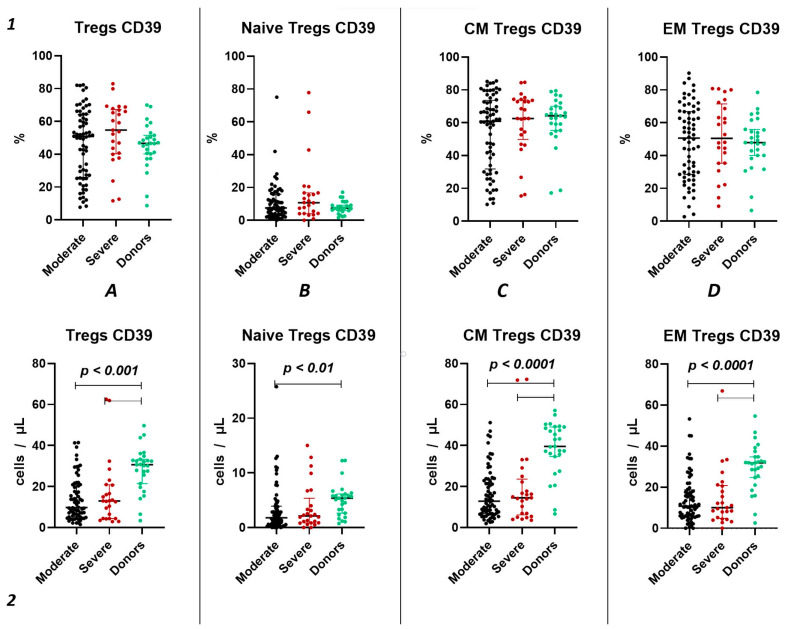
CD39 expression on T regulatory cell subpopulations in acute COVID-19. Upper row (**1**)—relative counts; bottom row (**2**)—absolute number. (**A**). CD39^+^ regulatory T cells. (**B**). CD39^+^ naïve regulatory T cells (Naïve Tregs). (**C**). CD39^+^ central memory regulatory T cells (CM Tregs). (**D**). CD39^+^ effector memory regulatory T cells (EM Tregs).

We did not observe significant differences in the relative count of CD39^+^ Tregs between both groups of patients with acute COVID-19 and the healthy donors group (Figure 3(1)). However, we observed a significant decrease in the absolute count of CD39^+^ total Tregs, central memory Tregs, and effector memory Tregs in both moderate and severe cases of acute COVID-19 compared to the healthy volunteers (Figure 3(2A,C,D)). Additionally, reduced levels of “naïve” CD39^+^ Tregs were evident in moderate COVID-19 patients when compared to the control group (Figure 3(2B)). Moreover, no significant differences were found between severity groups (Figure 3(2)).

Importantly, relative counts of CD39^+^ Tregs positively correlated with age (r = 0.29171), lung damage according of CT investigation (r = 0.30341), as well as levels of CRP (r = 0.34163), ferritin (r = 0.31563), and procalcitonin (r = 0.41087). Additionally, we found a negative correlation between relative counts of CD39^+^ CM Tregs and results of CT (r = 0.28195), levels of CRP (r = 0.27976), ferritin (r = 0.28380), and procalcitonin (r = 0.37262). Relative counts of CD39^+^ EM Tregs correlated with levels of D-dimer (r = 0.30399). Moreover, we observed a correlation between absolute counts of CD39^+^ Tregs and levels of LDG (r = 0.28092).

#### 2.3.2. Characteristics of CD73 Expression in Tregs in Acute COVID-19

**Figure 4:**
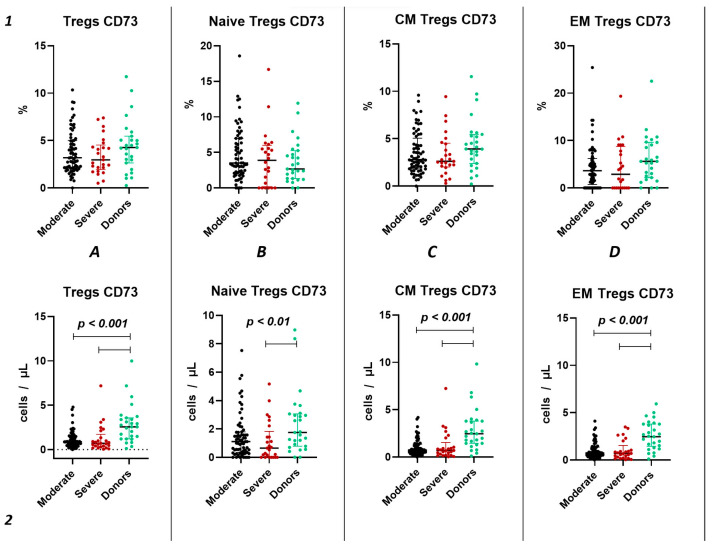
CD73 expression on T regulatory cell subpopulations in acute COVID-19. Upper row (**1**)—relative counts; bottom row (**2**)—absolute number. (**A**). CD73^+^ regulatory T cells. (**B**). CD73^+^ naïve regulatory T cells (Naïve Tregs). (**C**). CD73^+^ central memory regulatory T cells (CM Tregs). (**D**). CD73^+^ effector memory regulatory T cells (EM Tregs).

Similar to the analysis of CD39^+^ Tregs populations, we did not find significant changes in the relative content of CD73^+^ regulatory T lymphocytes and subsets in both groups of patients with acute COVID-19 and compared to the healthy donors group (Figure 4(1)). We also observed no difference in the relative content of CD73^+^ regulatory T lymphocytes between moderate and severe COVID-19 in all populations (Figure 4(1)). However, we observed a decrease in the absolute count of CD73^+^ Tregs, CM Tregs, and EM Tregs in moderate and severe COVID-19 patients compared to the healthy donors (Figure 4(2)). Furthermore, we observed a negative correlation between the absolute counts of CD73^+^ Naive Tregs and IL-1α (r = −0.83030), as well as between levels of CD73^+^ EM Tregs and age (r = −0.27245).

### 2.4. Tregs Subsets Restoration After COVID-19

**Figure 5:**
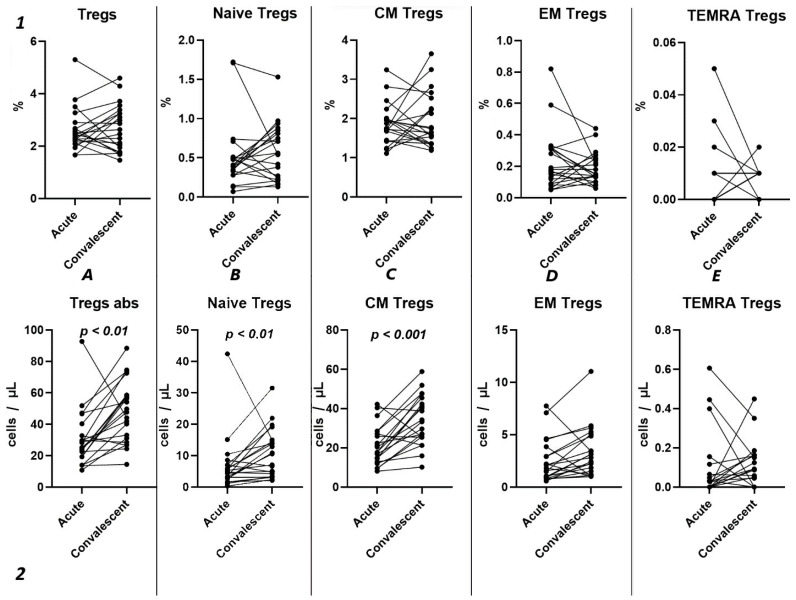
Dynamic changes in Tregs subsets. Upper row (**1**)—relative counts; bottom row (**2**)—absolute number. (**A**). Total T regulatory T cells (Tregs). (**B**). Naïve Tregs. (**C**). Central memory (CM) Tregs. (**D**). Effector memory (EM) Tregs. (**E**). Terminally differentiated CD45RA^+^ effector cells (TEMRA) Tregs.

Analyzing the cell-restoration process of each patient after 3–6 months after acute infection, we observed no significant dynamic changes in the relative counts (Figure 5(1)) of total Tregs (2.42% (2.15; 2.79) to 2.63% (1.86; 3.32)) and their subpopulations, including “naïve” Tregs (0.41% (0.31; 0.51) to 0.56% (0.23; 0.83)), CM (1.75% (1.43; 2.00) to 1.65% (1.36; 2.39)), EM Tregs (0.17% (0.09; 0.31) to 0.15% (0.12; 0.25)), and TEMRA Tregs (0.00% (0.00; 0.01) to 0.01% (0.00; 0.01)).

However, a significant increase was found in the levels of total Tregs (25.18 cells/μL (19.30; 36.49) to 47.72 cells/μL (29.83; 58.39)), as well as naïve (4.72 cells/μL (2.66; 7.13) to 10.50 cells/μL (3.79; 14.77)), and CM (17.85 cells/μL (13.71; 27.79) to 33.26 cells/μL (25.43; 43.86)).

**Figure 6:**
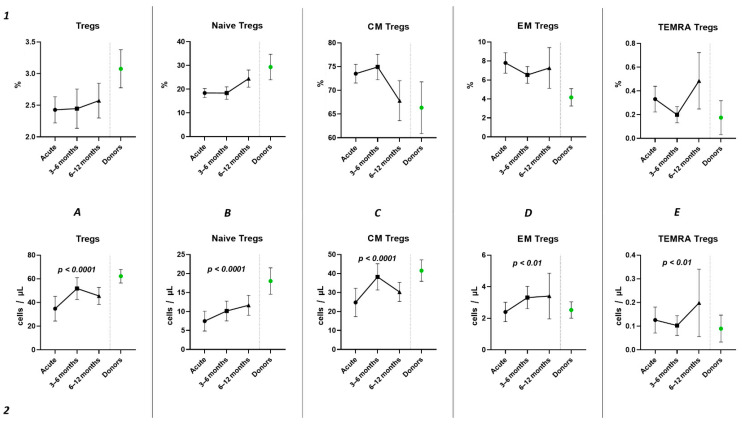
Three-point dynamic assessment of relative and absolute number of Treg subsets. Upper row (**1**)—relative counts; bottom row (**2**)—absolute number. (**A**). Total T regulatory T cells (Tregs). (**B**). Naïve Tregs. (**C**). Central memory (CM) Tregs. (**D**). Effector memory (EM) Tregs. (**E**). Terminally differentiated CD45RA^+^ effector cells (TEMRA) Tregs. Results presented as mean with 95% CI.

Subsequently, we conducted an examination of the progression dynamics of COVID-19, employing a three-point assessment through the application of the Kruskal–Wallis test. The initial assessment represented the acute phase of COVID-19, the second point reflected the condition 3–6 months post-convalescence, and the third point encompassed the convalescents 6–12 months after recovery. Evaluation of the relative values pertaining to T regulatory cells and their respective subpopulations revealed no statistically significant alterations in the observed dynamics (Figure 6(1)). The three-point dynamic assessment demonstrated a significant progressive gain of absolute numbers in total Tregs and all their subsets (Figure 6(2)). Nevertheless, even 6–12 months after infection, absolute counts of total Tregs (40.21 cells/μL (31.08; 55.18 to 66.91 cells/μL (49.23; 70.09)), Naïve Tregs (8.97 cells/μL (5.06; 15.01) to 15.97 cells/μL (11.16; 24.73)), and CM Tregs (27.44 cells/μL (19.86; 40.23) to 40.31 cells/μL (31.14; 51.29)) remained decreased compared to those of healthy donors.

#### 2.4.1. Dynamics of CD39 Expression in Peripheral Blood Regulatory T Lymphocytes

**Figure 7:**
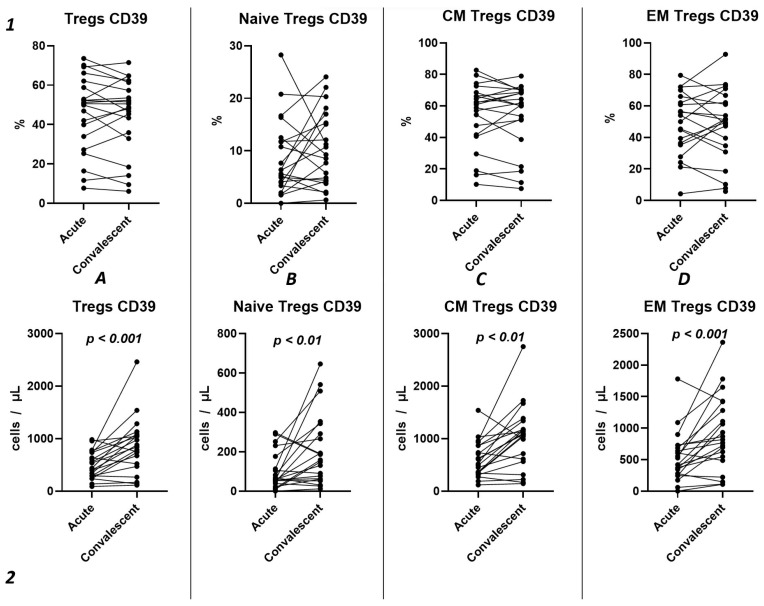
Dynamics of CD39^+^ T regulatory cell subpopulations. Upper row (**1**)—relative counts; bottom row (**2**)—absolute number. (**A**). Total T regulatory T cells (Tregs). (**B**). Naïve Tregs. (**C**). Central memory (CM) Tregs. (**D**). Effector memory (EM) Tregs.

Using the Wilcoxon test, we investigated the temporal changes in CD39 expression on Tregs and their subpopulations in a cohort of 21 patients, spanning from the acute phase of COVID-19 to the convalescent stage at 3–6 months (Figure 7). Our results show no statistically significant differences in the relative counts (Figure 7(1)) of CD39^+^ Tregs and subpopulations. We observed a significant increase in CD39 expression from the acute to the post-convalescent period in the total Tregs count (435.1 cells/μL (291.2; 736.9) and 841.1 cells/μL (495.3; 1068), respectively) (Figure 7(2A)) and in Treg subpopulations (Figure 7(2B–D)). This includes an increase in Naïve Tregs (57.51 cells/μL (42.91; 144.8) and 158.4 cells/μL (62.42; 317.6), respectively), CM Tregs (517.1 cells/μL (343.8; 865.2) and 1079 cells/μL (587.0; 1258), respectively), and EM Tregs (420.1 cells/μL (261.9; 705.9) and 817.8 cells/μL (516.5; 1350), respectively).

**Figure 8:**
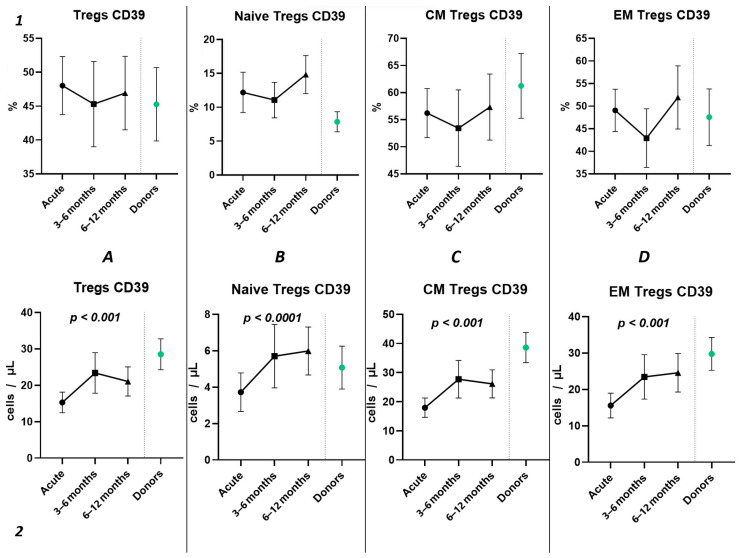
The three-point dynamic assessment of relative and absolute numbers of CD39^+^ Tregs and their subpopulations. Upper row (**1**)—relative counts; bottom row (**2**)—absolute number. (**A**). Total T regulatory T cells (Tregs). (**B**). Naïve Tregs. (**C**). Central memory (CM) Tregs. (**D**). Effector memory (EM) Tregs. Results presented as mean with 95% CI.

The three-point dynamic assessment demonstrated no statistically significant alterations in the relative values of T regulatory cells (Figure 8(1)) and a significant progressive increase in absolute counts of total Tregs and their subsets (Figure 8(2)). Nevertheless, even 12 months after infection, the levels of total Tregs (20.41 cells/μL (13.05; 24.66) to 30.65 cells/μL (21.43; 33.30)) and CM Tregs (16.19 cells/μL (9.21; 19.71) to 27.59 cells/μL (19.30; 30.98)), expressing CD39, remained decreased compared to those of healthy donors.

#### 2.4.2. Dynamics of CD73 Expression in Peripheral Blood Regulatory T Lymphocytes

**Figure 9:**
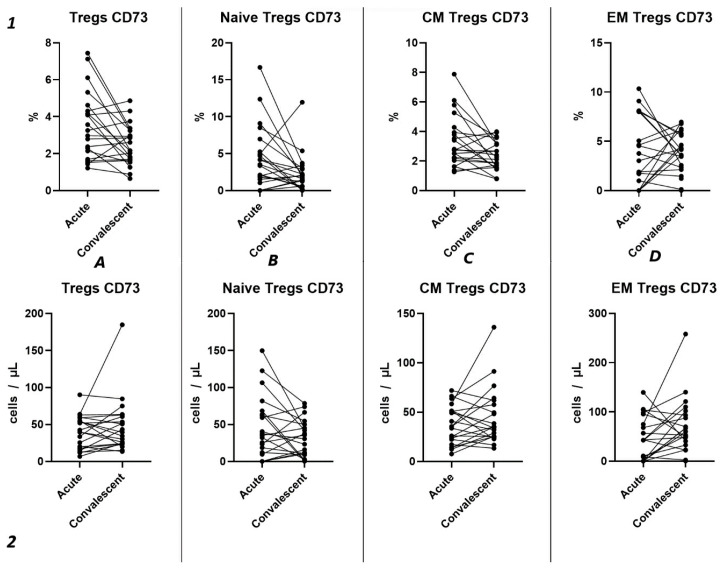
Dynamics of CD73^+^ T regulatory cell subpopulations. Upper row (**1**)—relative counts; bottom row (**2**)—absolute number. (**A**). Total T regulatory T cells (Tregs). (**B**). Naïve Tregs. (**C**). Central memory (CM) Tregs. (**D**). Effector memory (EM) Tregs.

Our findings reveal no significant differences in either the relative (Figure 9(1)) or absolute counts (Figure 9(2)) of CD73^+^ Tregs and all their subsets in dependent groups.

**Figure 10:**
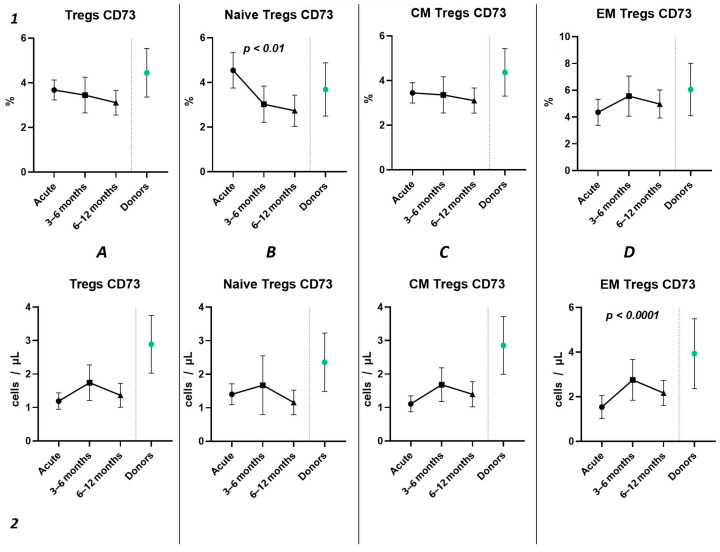
The three-point dynamic assessment of relative and absolute numbers of CD73^+^ Tregs and their subpopulations. Upper row (**1**)—relative counts; bottom row (**2**)—absolute number. (**A**). Total T regulatory T cells (Tregs). (**B**). Naïve Tregs. (**C**). Central memory (CM) Tregs. (**D**). Effector memory (EM) Tregs. Results presented as mean with 95% CI.

Three-point assessment of expression of CD73^+^ on Tregs demonstrated a significant progressive decrease in relative numbers of CD73^+^ Naïve Tregs (Figure 10(1B)) and significant increase in absolute counts of CD73^+^ EM Tregs (Figure 10(2D)). Moreover, even by 1 year after infection, the absolute counts of total Tregs (0.97 cells/μL (0.47; 1.72) to 2.56 cells/μL (1.21; 3.58)), Naïve Tregs (0.12 cells/μL (0.051;0.42) to 0.50 cells/μL (0.24; 0.79)), and CM Tregs (0.62 cells/μL (0.34; 1.08) to 1.29 cells/μL (0.84; 2.52)), expressing CD73, were decreased compared to healthy donors.

### 2.5. Cytokines Levels and Their Relationships with Levels of Immune Cells

**Figure 11:**
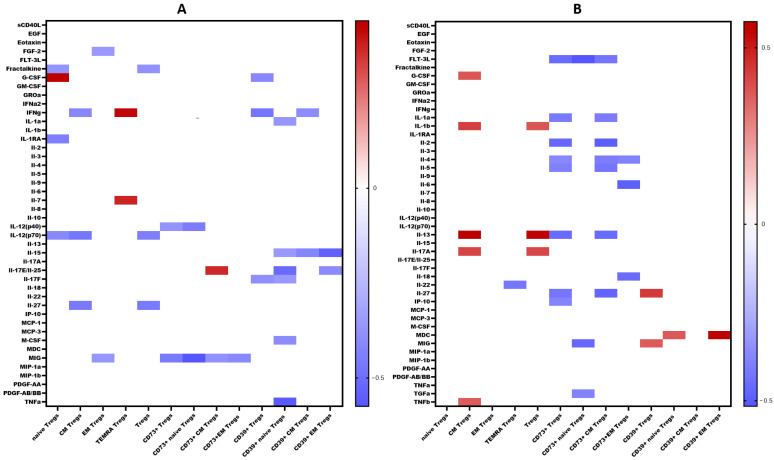
Heat map of correlations between cytokine levels and relative count of Treg subsets in convalescents after COVID-19 (**A**). At 3–6 months. (**B**). At 6–12 months. Color scale bar shows a range of correlation coefficients (r). The red color represents a high positive correlation, decreasing to the blue color bar, which represents a negative correlation. Heat map shows only significant correlation coefficients (*p* < 0.05).

We examined the levels of 47 cytokines, chemokines, and growth factors (IL-1α, IL-1β, IL-1 receptor antagonist (IL-1Ra), IL-2, IL-3, IL-4, IL-5, IL-6, IL-7, IL-9, IL-10, IL-12 (p40), IL-12 (p70), IL-13, IL-15, IL-17A/CTLA8, IL-17-E/IL-25, IL-17F, IL-18, IL-22, IL-27; chemokines CCL2/MCP-1, CCL3/MIP-1α, CCL4/MIP-1β, CCL7/MCP-3, CCL11/eotaxin, CCL22/MDC, CXCL1/GROα, CXCL8/IL-8, CXCL9/MIG, CXCL10/IP-10, CX3CL1/fractalkine; growth factors EGF, FGF-2/FGF-basic, Flt3 ligand, G-CSF, M-CSF, GM-CSF, PDGF-AA, PDGF-AB/BB, TGF-α, VEGF-A; and pro-inflammatory cytokines IFNα2, IFNγ, TNFα, TNFβ/lymphotoxin-α(LTA), and the soluble form of CD40L (sCD40L)) in the blood plasma of patients with COVID-19 during the acute phase of infection, as well as in the convalescent phase (3–6 months and 6–12 months post-recovery). The results are summarized in Table 1. A comprehensive analysis of cytokine levels during the acute phase has been previously reported [23]; thus, in this study, we focused on cytokine dynamics during the convalescent period. Correlations between cytokine levels and T regulatory cell subpopulations are depicted in Figure 11.

## 3. Discussion

Several studies have shown a decrease in T cell numbers in patients with COVID-19, which is associated with disease severity [24,25]. At the early stages of acute COVID-19, patients demonstrated a decline in CD3^+^ T cell count until recovery from SARS-CoV-2-induced pneumonia [26,27]. In our study, we examined the relative and absolute frequencies of CD3^+^ T lymphocytes and found correlations between circulating T cell levels in the peripheral blood and clinical characteristics in patients with acute COVID-19 (Figure 1E). Primarily, we noticed a significant decrease in the absolute counts of circulating T cells during the acute phase of SARS-CoV-2 infection. Similarly, multiple studies suggested that lymphocytopenia, including low CD3^+^ T cell levels, along with elevated inflammatory markers such as serum ferritin and high-sensitivity C-reactive protein, increased the risk of acute respiratory distress syndrome (ARDS) development and poor prognosis [28,29,30,31,32]. Our data also confirmed that the levels of CRP, ferritin, procalcitonin, and D-dimer were increased in patients with severe COVID-19. We noticed decreased frequencies of circulating CD3^+^CD4^+^ and CD3^+^CD8^+^ T cells.

Currently, the reports on the dynamics of absolute and relative frequencies of circulating Treg cells in patients with acute COVID-19 remain controversial. On the one hand, in patients with mild COVID-19, the absolute counts of CD3^+^CD4^+^CD25^+^CD127^low^ Tregs were elevated if compared to healthy control, whereas in patients with severe COVID-19 only relative numbers of Tregs were increased [9]. Additionally, patients with moderate and severe COVID-19 showed reduced expression of CD127 compared to the control group. However, it should be noted that the expression of this marker was normalized only in patients with mild infection upon recovery, whereas it remained reduced after recovery in severe cases [33]. Wang et al. observed an increase in the number of Tregs cells as patients with COVID-19 transitioned from mild to severe conditions, while circulating Tregs reduced during the progression from severe to critical condition state [12]. Additionally, it was noted that patients with severe COVID-19 had increased levels of CXCR3 on Tregs cells, indicating the upregulated capacities of Tregs to migrate to the sites of inflammation [13]. It was indicated that patients with severe COVID-19 exhibit elevated levels of CD25^+^FOXP3^+^ Tregs within total CD4^+^ T cells with increased FOXP3 expression, which normalized in recovering individuals or convalescent patients [16]. Moreover, Vick et al. found a rise in the number of CD25^+^CD127^+^FOXP3^+^ Tregs, accompanied with increased suppressive activity, in severe COVID-19 patients [20]. In contrast to healthy donors and COVID-19 convalescents, patients with acute SARS-CoV-2 infection exhibited a significant increase in the percentage of CD25^+^CD127^−^ Tregs within total Th cells subset. Interestingly, an increased number of Naïve Tregs (CD45RA^+^CCR7^+^) and central memory Tregs (CD45RA^−^CCR7^+^) with strong PD-1 expression were reported in patients with COVID-19 [34]. In addition, an increase in central memory Tregs with elevated expression of CTLA-4 and IL-10 was observed in patients with mild, moderate, and severe COVID-19 [20].

On the other hand, numerous studies have reported a decrease in the number of Tregs in patients with COVID-19. For instance, Mahmoud Salehi Khesht et al. found a substantially enhanced Th17/Treg ratio and a significant reduction in Tregs in ICU-hospitalized patients [35]. Another study observed a similar rise in the Th17/Treg ratio in PBMCs from patients with COVID-19, associated with negative outcomes and lower levels of TGF-β and IL-10 [14]. A comparative analysis reported decreased levels of Tregs in patients with severe COVID-19 if compared to patients with mild symptoms [36]. Furthermore, individuals with severe COVID-19 were found to have a lower percentage of CD3^+^CD25^+^ Tregs [29]. Interestingly, Tregs were decreased during the acute phase of SARS-CoV-2 infection in children but returned to baseline after recovery [37]. Moreover, several studies indicated a tendency toward low levels of CD25^+^FoxP3^+^CD127^–^ Tregs in peripheral blood even after recovery [11]. In our study (Figure 1D), we found that patients with acute COVID-19 in both groups with different COVID-19 severities had a decrease in the relative and absolute numbers of Tregs, and, of note, their frequencies correlated with procalcitonin levels (Figure 1E). The inverse correlation with procalcitonin may suggest an increased likelihood of sepsis development with a reduction in the levels of regulatory T cells.

In the study by Yang et al., two main Treg subsets were identified, including resting cells (nTregs, CD45RA^+^FoxP3^low^) and activated T regulatory cells (aTregs, CD45RA^-^FoxP3^hi^) [38]. The observations showed a four-fold increase in aTregs cells in patients with COVID-19, especially in the early stages of the disease. Activated Tregs were characterized by high CTLA-4 expression, which contributes to the transmission of inhibitory signals to T cells. To analyze the impact of SARS-CoV-2 on different types of regulatory T cells, we identified four Tregs subsets: “naïve” or “thymic” Tregs (with the CD45RA^+^CD62L^+^ phenotype), central and effector memory Tregs (with the CD45RA–CD62L^+^ and CD45RA^–^CD62L^–^ phenotypes, respectively), and terminally differentiated effector Tregs (phenotype CD45RA^+^CD62L^–^). We observed a decrease in the absolute number of Naïve and central memory Tregs during acute COVID-19 (Figure 2A,B). Interestingly, increased relative numbers of effector memory Tregs were previously noticed in patients with moderate and severe acute SARS-CoV-2 infection (Figure 2C) [39]. A diminished level of Naïve Tregs correlated with indicators of systemic inflammatory response, such as CRP, ferritin, procalcitonin, and LDH. Previously, Rosichini et al. demonstrated that SARS-CoV-2 could target human thymic epithelial cells and reduce thymic functions, resulting in altered intrathymic T cell maturation [40]. Furthermore, altered emigration of mature T cells out of the thymus in patients with COVID-19 was associated with the severity and outcome of the disease [41]. This suggests a direct association of impaired Treg subsets with the development of a systemic inflammatory response. Additionally, Perfilyeva et al. found that Tregs exhibited a negative correlation with the cytokine M-CSF, which, in conjunction with Tregs-produced proinflammatory cytokines (IL-10 and TGFβ), is responsible for the differentiation of monocytic MDSCs and the suppression of an excessive immune response [39]. The obtained data indicate an intensive redistribution of Treg subsets. Specifically, the concentration and percentage of Naïve Tregs decrease, that may lead to impaired suppression of hyperinflammatory reactions and contribute to the development of autoimmune diseases, provoking the development of “post-COVID-19” syndrome.

Currently, the data on alterations in Treg cell subsets in SARS-CoV-2 convalescents are still limited [42]. Rajamanickam et al. demonstrate that Naïve CD4^+^T cells, regulatory T cells, transitional memory, and stem cell memory T-cell frequencies decreased from days 15–30 to days 61–90 and then remained steady [43]. One longitudinal study revealed a persistent decrease in the relative frequency of total Tregs from two months post-infection to the eight-month follow-up [44]. Similarly, an increase in “naïve” Tregs from the first (14–90 days post-infection) to the second (91–180 days post-infection) follow-up was observed, regardless to the initial disease severity, whereas central memory Tregs decreased. Another study indicated that increased Treg frequency in acute COVID-19 reduced during the recovery period to an equilibrium point [45]. Controversially, Ryan et al. demonstrated reduced levels of Tregs in acute COVID-19 and an increase at all time points post-infection [46]. In our study, analyzing paired data points from 21 patients, we observed a trend toward the restoration of decreased absolute numbers of Tregs and their distinct subsets, such as Naïve and central memory regulatory T lymphocytes, by 3–6 months post-acute COVID-19. Moreover, their frequencies remained unchanged over the six-month period. In a three-point dynamic assessment over a year, we did not detect alterations in the relative values of both Tregs and all their subpopulations. However, we identified a dynamic trend toward the restoration of absolute counts of Tregs and all their subsets. Notably, a positive restoration trend throughout the entire period was observed in “naïve” and effector memory Tregs. CM Tregs increased to donors’ levels by six months and subsequently declined to a level significantly different from the donors’ level by one year. TEMRA Tregs in peripheral blood increased 12 months post-recovery. It is worth noting that, despite the increase in Naïve Tregs, they did not reach the donors’ level by 12 months. Thus, impaired thymocyte selection, alterations in “natural” or “thymic” Treg maturation, as well as decreased levels of circulating Tregs in peripheral blood point to the prolonged Treg dysregulation in COVID-19 convalescents and may contribute to a post-COVID-19 associated immunopathology, including different autoimmune diseases.

Importantly, Tregs exhibit CD39 expression and play a role in pro-inflammatory ATP to AMP metabolism, thereby impeding dendritic cells maturation by depleting ATP [47]. Additionally, the co-expression of CD39 and CD73 on Tregs facilitates the conversion of ADP to anti-inflammatory adenosine. Adenosine, coupled with the adenosine A2A receptor on effector T cells, hampers their activation [48,49]. Currently, there is a lack of original data on the impact of coronavirus infection on the expression of purinergic signaling receptors on T lymphocytes. For instance, in the study by Parimah Ahmadi et al., it was reported that there were no differences in the expression of CD73 and CD39 on CD4^+^ T cells during coronavirus infection, although the level of CD73 on CD8^+^ T cells was reduced [50]. The authors note that COVID-19 was associated with decreased expression of CD73 on the surface of CD8^+^ T cells, which correlated with the serum ferritin levels, whereas the frequency of CD39, on the contrary, increased [50]. Similarly, in one study, cell type-specific analysis revealed higher frequencies of CD39^+^ T cells in severe COVID-19 patients, while the expression of CD73 on CD4^+^ and CD8^+^ cells was reduced in the same group [51]. Patients with mild COVID-19 exhibited reduced frequencies of CD4^+^CD25^+^CD39^+^ (activated/memory regulatory T cell [mTreg]) cells. In patients with severe COVID-19, decreased frequencies of CD4^+^CD73^+^, CD4^+^CD25^+^CD39^+^ mTreg cells, and CD8^+^CD73^+^ were noticed if compared to both mild COVID-19 patients and controls. Furthermore, plasma samples from severe COVID-19 patients demonstrated the capability to reduce the expression of CD73 on CD4^+^ and CD8^+^ T cells from a healthy donor [52]. Another study showed augmented CD39 expression in Treg (CD4^+^CD25^+^Foxp3^+^) compared to healthy donors [53]. It was also observed that, at the point of hospital discharge, the frequency of CD39^+^CD8^+^ T-cells significantly decreased, whereas the percentage of CD73^+^ cells increased [54]. Supporting these data, Alaa Elsaghir et al. reported an increased frequency of CD39^+^ Treg subsets in patients with severe COVID-19 compared to both healthy donors and patients with moderate disease [55]. Additionally, it was observed that serum adenosine levels were significantly decreased in these patients, particularly those with severe illness, which aligns with the marked decline in CD73 expression levels [55]. Furthermore, they noted that elevated levels of immunosuppressive cytokines, such as IL-10 and TGF-β, were associated with more severe disease manifestations [55].

In our study, we conducted a detailed analysis of purinergic signaling receptors (CD39 and CD73) expression on Tregs and their distinct subsets. Although we did not observe changes in the relative values of CD39^+^ and CD73^+^ in both the overall pool of Tregs and their subpopulations during the acute phase of COVID-19 infection, we noted alterations in absolute values. Specifically, we identified a decrease in the expression of both CD39 and CD73 in both severity groups of COVID-19 during the acute infection compared to healthy donors (Figure 5(2A) and Figure 6(2A)). The reduction in CD73 expression occurred in the CM and EM Tregs (Figure 4(2C,D)). Interestingly, the CD73^+^ Naïve Tregs level decreased only in the severe group, whereas it remained unchanged in the moderate severity group compared to the control group (Figure 4(2B)). Moreover, levels of CD73^+^ Naïve Tregs negatively correlated with proinflammatory cytokine IL-1α, pointing to alterations in Tregs cell maturation in thymus and to the importance of Naïve Tregs in controlling inflammation. Similarly to CD73, CD39 expression decreased in both severity groups on CM and EM Tregs (Figure 3(2C,D)). However, the reduction in expression on naive Tregs occurred only in the group of moderate COVID-19 (Figure 3(2B)). The total CD39^+^ Tregs and CD39^+^ CM Tregs demonstrated significant informativeness, as they exhibited positive correlations with biochemical markers of systemic inflammatory response, including CRP, ferritin, and procalcitonin. Particularly interesting, these cell populations also correlated with the extent of involvement identified through computed tomography results. Leveraging this information, these Treg subsets may serve as valuable indicators of the altered immunoregulatory CD39/CD73 axis during other viral infections [56] and autoimmunity [57].

Among other findings, we have described the dynamics of purinergic signaling receptors changes on different Treg subsets following recovery from COVID-19. When assessing paired data points from 21 patients, we did not observe significant changes in CD73 expression six months after acute COVID-19, both in absolute and relative counts (Figure 9). However, in a three-point dynamic assessment, a decrease was noted in the relative values of CD73^+^ Naïve Tregs (Figure 10(1B)). Additionally, there was a positive trend in the absolute values of CD73^+^ effector memory Tregs (Figure 10(2D)). In contrast, in paired comparisons, an increase in the absolute values of all CD39^+^ Tregs was observed at 6 months post-recovery, whereas there was no observable dynamic change in relative values (Figure 7). A similar pattern was demonstrated in the three-point dynamic assessment. The relative values of all CD39^+^ Treg subsets remained unchanged (Figure 8(1)). However, their absolute values increased dynamically over the course of a year after acute infection (Figure 8(2)). It is noteworthy that the overall pool of CD39^+^ regulatory T cells, despite the trend toward elevation, remained significantly lower than that of the healthy donors (Figure 8(2A)). This reduction is primarily attributed to CM Tregs, that also did not recover to the level of healthy donors (Figure 8(2C)). However, the absolute counts of CD39^+^ Naïve and EM Tregs reached levels in a year that were not significantly different from those of donor levels (Figure 8(2B,D)).

Interestingly, CD73 expression on convalescents correlated with MIG, a chemokine which is thought to be involved in T cells recruitment to the site of infection and virus elimination. We observed a negative correlation between MIG and CD73^+^ regulatory T cells, including both Naïve’ and CM subsets, as well as a positive correlation between MIG and CD39^+^ Tregs, including CD39^+^ CM Tregs. Furthermore, in recovered individuals, a reverse relationship was found between IL-2 and CD73^+^ Tregs and CD73^+^ EM Tregs. As is known, IL-2 is involved in the Th1-type immune response and the immune reaction against viral infections [58]. IL-2 signaling is essential for the generation of Th1 cells specific to viral antigens in the lung following infection. Additionally, it is necessary for the survival and effector functions of Tregs [59]. Moreover, CD39 expression on Naïve Tregs in convalescents correlated with IL-15, IL-17E/IL-25, TGF-α, and M-CSF. IL-15 is associated with promoting Th1 responses, characterized by the activation of CD8^+^ cytotoxic T cells, NK-cells with their production of interferon-gamma, as well as maintaining memory CTL [60]. It is intriguing that a correlation with IL-17E was observed primarily associated with the immune response to parasites and allergies [61]. However, it is also capable of inducing airways hyperactivity and development of virus-associated immunopathology [62,63]. Moreover, IL-17E may work in conjunction with TGF-α and M-CSF, promoting macrophage differentiation and recruitment, followed by their differentiation into alternatively activated macrophages (M2) that contribute to tissue healing and repair [64,65,66,67], in particular after viral infection and pneumonia.

**Limitations.** The majority of patients in the study were over 50 years of age. Due to the limited availability of individuals in this age group without any chronic conditions, assembling a healthy donor group was challenging. This contributed to significant differences in the median age between the patients and control group.

## 4. Materials and Methods

### 4.1. Patient Characteristics

This study included 93 patients (48 men and 45 women) with acute coronavirus infection complicated by pneumonia, admitted to the infectious diseases department of the Almazov National Medical Research Centre. All patients were hospitalized 5–7 days after the onset of the illness. The post-COVID cohort comprised 40 patients within the 3–6-month convalescent phase (23 men and 17 women) and 48 patients within the 6–12-month convalescent phase (25 men and 23 women). A comparison group included 27 healthy donors (14 men and 13 women). The diagnosis of COVID-19 in all patients was established based on epidemiological and clinical data. Computer tomography of the chest organs was performed to assess the extent of lung involvement in all patients. The diagnosis of coronavirus infection was confirmed by detecting SARS-CoV-2 RNA in nasal and nasopharyngeal swabs. A PCR-based SARS-CoV-2/SARS-CoV detection kit (DNA Technology, Moscow, Russia) was used for this purpose. Additionally, the level of total antibodies in the blood plasma was determined using the DS-ELISA-anti-SARS-CoV-2 kit (Diagnostic Systems, Nizhny Novgorod, Russia) for confirmation of the diagnosis.

The presence and variety of comorbidities in patients are presented in Table 2.

The severity of the disease was determined based on the extent of lung involvement as assessed by computed tomography (CT) scans and C-reactive protein (CRP) level (<50% CT involvement and CRP < 110)—moderate course; ≥50% CT involvement and CRP ≥ 110- severe course). No patients in the cohort died. The clinical and laboratory characteristics of patients at admission and healthy donors are presented in Table 3. A limitation of the study was the younger age of the control group compared to the patients. Lymphopenia was observed in all patients. Additionally, patients had lower levels of eosinophils and basophils in peripheral blood compared to donors. However, in both groups, these values were within the reference range.

Several peripheral blood parameters reflected the severity of the patients’ condition with elevated levels of C-reactive protein (CRP), ferritin, and lactate dehydrogenase (LDH) compared to normal values.

Patients were scheduled for follow-up appointments at 3–6 months and 6–12 months post-recovery. All patients underwent a clinical examination by a general practitioner and had a clinical blood test performed.

### 4.2. Sample Collection

Upon admission, all patients provided informed consent to participate in the study. The study was approved by the local ethics committee of the Almazov National Medical Research Centre (protocol no. 2209-20 dated 21 September 2020). All research was conducted in accordance with the Helsinki Declaration of the World Medical Association, “Ethical Principles for Medical Research Involving Human Subjects”. Blood samples from patients in the acute phase of COVID-19 and after recovery were collected and proceed within the first 24 h.

### 4.3. Antibodies and Flow Cytometry

The immune cell subsets were analyzed using multicolor flow cytometry. A peripheral blood sample was stained using FITC-labelled mouse anti-human CD39 (clone A1, cat. 328206, BioLegend, Inc., San Diego, CA, USA), PE-labelled mouse anti-human CD25 (clone B1.49.9, cat. A07774, Beckman Coulter, Indianapolis, IN, USA), PerCP/Cy5.5-labelled mouse anti-human CD45RA (clone HI100, cat. 304122, BioLegend, Inc., San Diego, CA, USA), PE/Cy7-labelled mouse anti-human CD4 (clone T4, cat 737660, Beckman Coulter, Indianapolis, IN, USA), APC-labelled mouse anti-human CD8 (clone B9.11, cat. IM2469, Beckman Coulter, Indianapolis, IN, USA), APC/Cyanine7-labelled mouse anti-human CD3 (clone OKT3, cat. 317342, BioLegend, Inc., San Diego, CA, USA), Pacific Blue-labelled mouse anti-human CD73 (clone AD2, cat. 344012, BioLegend, Inc., San Diego, CA, USA), ECD-labelled mouse anti-human CD62L (clone DREG56, cat IM2713U, Beckman Coulter, Indianapolis, IN, USA), and Krome Orange-labelled mouse anti-human CD45 (clone J33, cat. A96416, Beckman Coulter, Indianapolis, IN, USA).

The detailed sample preparation and analysis has been described earlier [68,69]. In brief, the aforementioned panel of monoclonal antibodies was used to stain 100 μL of peripheral blood according to the manufacturer’s recommendations. Erythrocyte removal from the samples was performed using a no-wash technique with the lysis solution VersaLyse (cat. no. A09777, Beckman Coulter, Indianapolis, IN, USA), to which 975 μL ex tempore of fixative solution IOTest 3 Fixative Solution (cat. no. A07800, Beckman Coulter, Indianapolis, IN, USA) was added. After erythrocyte lysis, the samples were washed once with an excess of physiological saline solution at 330 g for 7 min. The supernatant was then removed, and the cell pellet was resuspended in a physiological saline solution with a pH of 7.2–7.4 containing 2% paraformaldehyde (Sigma-Aldrich, St. Louis, MO, USA). Sample analysis was performed on a CytoFlex S flow cytometer (Beckman Coulter, Brea, CA, USA) equipped with four diode lasers at 405, 488, 561, and 638 nm. At least 60,000 CD3^+^ T lymphocytes from peripheral blood were analyzed in each sample.

### 4.4. Detection of Major Subpopulations of T Lymphocytes

**Figure 12:**
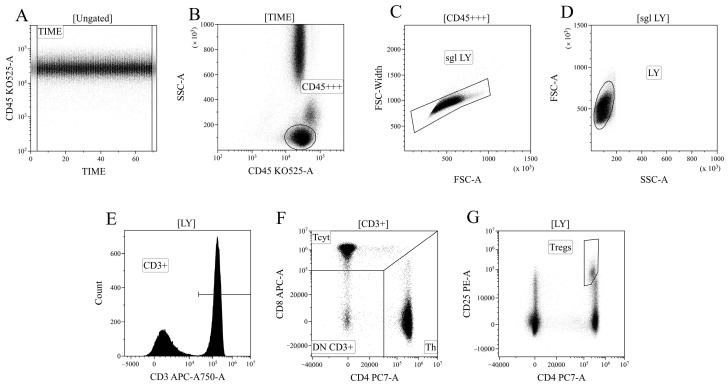
Consistent gating strategy for regulatory T-cells phenotyping. (**A**). Initial gating of time of stable flow. (**B**). Identification of lymphocytes, based on CD45 expression. (**C**). Exclusion of cell aggregates. (**D**). Identification of lymphocytes, based on their morphology. (**E**). Gating T-cells, expressing CD3. (**F**). Gating of helper and cytotoxic T-cells, based on their expression of CD4 and CD8, respectively. (**G**). Identification of regulatory T-cells with CD3^+^CD4^+^CD25^bright^ phenotype.

Data processing of cytometric data was performed using the Kaluza™ v.2.3 software (Beckman Coulter, Brea, CA, USA). In the initial stage of analysis (Figure 12A), a time segment with stable data collection conditions, where cell analysis was performed, was selected based on the “CD45 expression versus analysis time” histogram. Next (Figure 12B), based on ^bright^ expression of CD45 (*x*-axis) and low values of the SSC parameter, which characterizes cell structure (*y*-axis), the lymphocyte population was identified (region “CD45^+++^”). Then, (Figure 12C), using the analysis of linear values of the integral FSC parameter (characterizing relative cell size, *x*-axis) and the time it takes for cells to intersect the laser beam (FSC–W, time of flight, *y*-axis), single non-aggregated lymphocytes were identified (region “sgl LY”). Finally, morphological characteristics were analyzed (Figure 12D)—relative cell size (*y*-axis, FSC) along with the complexity of their cytoplasmic organization (*x*-axis, SSC). Using the histogram characterizing the expression of CD3, CD3-positive T lymphocytes in peripheral blood were identified (Figure 12D). Within the overall pool of T cells, based on the analysis of CD4 and CD8 expression, T helper cells and cytotoxic T lymphocytes with CD3^+^CD4^+^ and CD3^+^CD8^+^ phenotypes, respectively, were identified (Figure 12F). Additionally, within the overall pool of lymphocytes, regulatory T lymphocytes (Tregs) with the CD3^+^CD4^+^CD25^bright^ phenotype were identified, as shown in Figure 12G.

**Figure 13:**
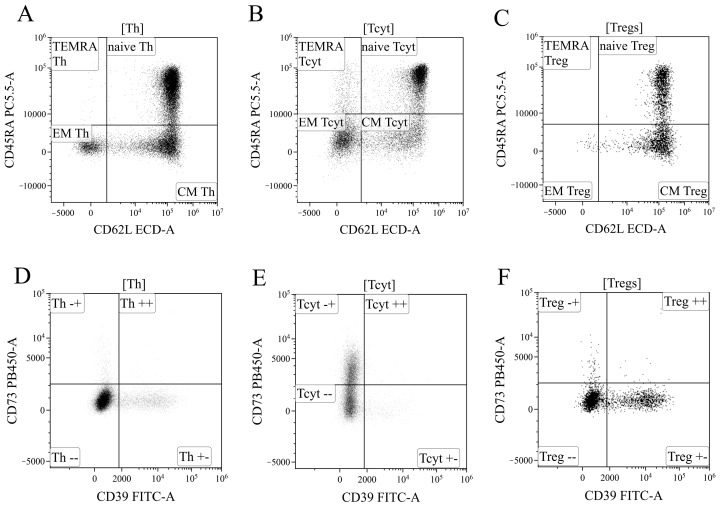
Gating strategy for regulatory T-cells subsets phenotyping. (**A**–**C**). Gating of naïve, central memory (CM), effector memory (EM), and T effector memory re-expressing CD45RA (TEMRA), based on their expression of CD45RA and CD62L, in helper (Th), cytotoxic (Tcyt) and regulatory T-cells (Tregs), respectively. (**D**–**F**). Expression of CD73 and CD39 on helper, cytotoxic, and regulatory T-cells, respectively.

Based on the analysis of CD45RA and CD62L expression, the overall pool of T helper cells was divided into four key cell subpopulations, including “naïve” Th cells with the CD45RA^+^CD62L^+^ phenotype, central and effector memory cells (“CM Th” and “EM Th”, A, with CD45RA–CD62L^+^ and CD45RA–CD62L–phenotypes, and “terminally differentiated” CD45RA^+^ effector cells (TEMRA population with the CD45RA^+^CD62L–phenotype)) (Figure 13A). Similarly, cytotoxic T lymphocytes and Tregs were divided into distinct subpopulations (Figure 13B,C). The subdivision of Tregs into separate subtypes was based on the following studies [70,71,72].

Finally, using the histograms displaying the distribution of T cells based on the expression of ectonucleoside triphosphate diphosphohydrolase-1 CD39 (*x*-axis) and ecto-5′-nucleotidase CD73 (*y*-axis) (Figure 13), all the previously identified cell populations were analyzed, including the overall T helper cells (Figure 13D), CD8^+^ T cells (Figure 13E), and Tregs (Figure 13F), as well as the populations of “naïve cells, central memory cells, effector memory cells, and terminally differentiated effector cells within the aforementioned T lymphocyte populations.

### 4.5. Cytokines Multiplex Analysis

The quantification of 47 cytokines/chemokines/growth factors (IL-1α, IL-1β, IL-1 receptor antagonist (IL-1Ra), IL-2, IL-3, IL-4, IL-5, IL-6, IL-7, IL-9, IL-10, IL-12 (p40), IL-12 (p70), IL-13, IL-15, IL-17A/CTLA8, IL-17-E/IL-25, IL-17F, IL-18, IL-22, IL-27; chemokines CCL2/MCP-1, CCL3/MIP-1α, CCL4/MIP-1β, CCL7/MCP-3, CCL11/eotaxin, CCL22/MDC, CXCL1/GROα, CXCL8/IL-8, CXCL9/MIG, CXCL10/IP-10, CX3CL1/fractalkine; growth factors EGF, FGF-2/FGF-basic, Flt3 ligand, G-CSF, M-CSF, GM-CSF, PDGF-AA, PDGF-AB/BB, TGF-α, VEGF-A; and pro-inflammatory cytokines IFNα2, IFNγ, TNFα, TNFβ/lymphotoxin-α(LTA), and soluble form of CD40L (sCD40L)) in plasma samples was conducted through multiplex analysis using the MILLIPLEX^®^ MAP Human Cytokine/Chemokine/Growth Factor Panel A (HCYTA-60K-PX48, MilliporeSigma, Burlington, MA, USA) on the Luminex MAGPIX^®^ (RUO) Instrument (Luminex, Austin, TX, USA), following the manufacturer’s instructions and as was previously performed by our group [23,73]. Briefly, 25 µL of plasma samples and fluorescently labeled magnetic microsphere beads were added in appropriate wells and incubated on a plate shaker overnight at +4 °C in the dark. Subsequent steps included washing, addition of detection antibodies with incubation at room temperature in the dark for 1 h, and incubation with streptavidin–phycoerythrin under the same conditions. Standard samples were prepared per the manufacturer’s recommendations, and calibration curves were established for each analyte. Data acquisition utilized xPONENT software (Version 4.3), and analysis was performed using Milliplex Analyst 5.1 Flex software.

### 4.6. Statistical Analysis

The sample description was performed by calculating the median (Me) and the interquartile range represented by the first and third quartiles (Q25; Q75). The significance of differences between independent sample measurements was evaluated using the non-parametric Kruskal–Wallis test, adjusted for multiple comparisons using the Dunn correction. The differences between donors and patients were estimated using Mann–Whitney U-test. The differences between dependent groups during the time were analyzed using the Wilcoxon test. To investigate the strength of correlations between variables, the Spearman rank correlation coefficient (Spearman rank R) was calculated. Statistical analysis was conducted using the software packages Statistica 12.0 (StatSoft Inc., Tulsa, OK, USA, 2013) and GraphPad Prism 8 (GraphPad software Inc., San Diego, CA, USA). Differences were considered significant at *p* < 0.01.

## 5. Conclusions

Our study revealed a significant decrease in the proportion and absolute number of Tregs during acute SARS-CoV-2 infection in patients with moderate and severe COVID-19. Even 6–12 months post-infection, the absolute number of Tregs remained decreased compared to healthy controls. We observed a reduction in the relative number of naïve Tregs and an increase in effector memory (EM) Tregs, along with a decrease in the absolute number of naïve and central memory (CM) Tregs. During the early convalescent period, the absolute counts of all Treg populations tended to increase, accompanied by a reduction in pro-inflammatory cytokines. However, one year post-recovery, the decreased Treg cell subsets had not yet reached the levels observed in healthy donors. This persistent imbalance in Treg subsets and impaired Treg function (CD39 and CD73 expression, and associated signaling pathways) may contribute to the impaired suppression of hyperinflammatory reactions, potentially leading to the development of autoimmune diseases and “post-COVID-19” syndrome. Understanding these alterations in Tregs and the ATP/adenosine signaling pathway offers novel insights into the pathogenesis of “post-COVID-19” syndrome and could be crucial in developing novel therapeutic approaches.

## Figures and Tables

**Table 1:** Plasma cytokine levels in patients with acute COVID-19 and convalescents.

Cytokine	Acute COVID-19	3–6 Months	6–12 Months
sCD40L, pg/L	6877 (2325; 9681)	1228 (576; 3938) ^	499 (313; 1585) *^
EGF, pg/L	15.5 (9.7; 40.5)	0.0 (0.0; 19.2)	0.0 (0.0; 11.1) *
Eotaxin, pg/L	143.0 (104.7; 196.6)	103.5 (88.1; 132.5)	107.5 (72.5; 136.3)
FGF-2, pg/L	52.9 (0.0; 65.6)	61.6 (0.0; 82.5)	49.8 (0.0; 76.7)
LT-L, pg/L	9.5 (5.1; 16.9)	16.1 (8.1; 21.8)	8.4 (5.1; 18.1)
Fractalkine, pg/L	136.1 (125.7; 250.2)	164.6 (134.1; 222.2)	164.6 (134.1; 191.6)
G-CSF, pg/L	53.0 (16.7; 64.6)	0.0 (0.0; 0.0) *	0.0 (0.0; 20.6) *
GM-CSF, pg/L	0.0 (0.0; 0.0)	0.0 (0.0; 0.0)	0.0 (0.0; 0.0)
GROa, pg/L	10.6 (0.0; 29.5)	0.0 (0.0; 3.9) *	0.0 (0.0; 0.0) *
IFNa2, pg/L	81.5 (53.9; 129.3)	49.5 (39.9; 70.3) *	49.5 (29.2; 66.4) *
IFNg, pg/L	9.0 (3.7; 14.3)	0.0 (0. 0; 3.2) *	0.0 (0.0; 3.2) *
IL-1a, pg/L	8.6 (6.5; 11.5)	9.8 (6.5; 19.2)	10.7 (5.6; 23.4)
IL-1b, pg/L	12.0 (8.2; 21.2)	14.7 (4.2; 31.3)	8.7 (0.5; 20.4)
IL-1RA, pg/L	6.4 (4.8; 30.1)	3.9 (2.5; 6.7) *^	2.1 (1.4; 3.5) *^
IL-2, pg/L	0.0 (0.0; 1.0)	0.0 (0.0; 1.5)	0.0 (0.0; 1.2)
IL-3, pg/L	0.1 (0.0; 0.4)	0.0 (0.0; 0.1)	0.0 (0.0; 0.0)
IL-4, pg/L	0.0 (0.0; 4.4)	0.0 (0.0; 1.6)	0.0 (0.0; 1.3)
IL-5, pg/L	3.2 (1.9; 10.5)	3.3 (2.1; 5.7)	3.1 (2.2; 4.7)
IL-6, pg/L	11.1 (5.7; 27.2)	2.2 (0.9; 3.0) *	1.7 (0.9; 2.9) *
IL-7, pg/L	2.7 (1.1; 3.8)	0.8 (0.0; 2.4)	0.8 (0.0; 1.8) *
IL-8, pg/L	9.1 (6.4; 19.2)	4.8 (3.2; 7.0) *^	3.5 (1.8; 5.1) *^
IL-9, pg/L	0.0 (0.0; 12.4)	0.0 (0.0; 11.7)	0.0 (0.0; 11.5)
IL-10, pg/L	18.1 (15.0; 36.9)	0.0 (0.0; 0.0) *	0.0 (0.0; 0.0) *
IL-12 p40, pg/L	61.5 (49.2; 81.8)	40.8 (28.6; 64.5)	37.2 (27.4; 53.9)
IL-12 p70, pg/L	3.2 (2.3; 5.2)	3.8 (2.1; 5.7)	2.5 (0.7; 4.6)
IL-13, pg/L	40.9 (0.0; 70.5)	46.5 (31.2; 135.2)	39.1 (0.0; 76.9)
IL-15, pg/L	27.4 (22.3; 30.6)	14.5 (11.1; 16.7) *	11.9 (8.8; 15.6) *
IL-17A, pg/L	5.0 (0.0; 7.2)	6.0 (0.0; 11.2)	5.0 (0.0; 8.2)
IL-17E/IL-25, pg/L	371.6 (302.6; 448.8)	213.9 (143.3; 324.1) *	236.5 (131.0; 366.4)
IL-17F, pg/L	0.0 (0.0; 14.5)	0.0 (0.0; 0.0)	0.0 (0.0; 0.0)
IL-18, pg/L	115.7 (45.5; 217.3)	41.1 (29.7; 77.4) ^	28.5 (16.8; 41.9) *^
IL-22, pg/L	0.0 (0.0; 0.0)	0.0 (0.0; 0.0)	0.0 (0.0; 0.0)
IL-27, pg/L	3207 (1920; 3924)	1487 (857; 2195) *	1731 (1262; 2488)
IP-10, pg/L	4425 (1532; 7602)	287 (208; 418) *	217 (141; 328) *
MCP-1, pg/L	729.0 (513.7; 813.2)	328.4 (288.3; 502.2) *	355.1 (281.1; 453.5) *
MCP-3, pg/L	28.0 (21.7; 37.8)	25.0 (21.7; 35.6)	24.0 (14.7; 28.9)
M-CSF, pg/L	249.6 (159.9; 302.1)	0.0 (0.0; 58.0) *	36.3 (0.0; 61.4) *
MDC, pg/L	657.6 (487.7; 814.2)	707.2 (606.5; 861.1)	603.5 (501.2; 761.4)
MIG, pg/L	2899 (2075; 3193)	1440 (1118; 2545)	1857 (1044; 2821)
MIP-1a, pg/L	23.2 (20.1; 26.1)	23.8 (16.0; 33.4)	17.4 (0.0; 28.3)
MIP-1b, pg/L	36.6 (21.8; 60.0)	25.4 (21.4; 33.5)	27.2 (20.2; 35.5)
PDGF-AA, pg/L	2050 (1496; 4252)	1597 (906; 2474)	1260 (518; 2262)
PDGF-AB/BB, pg/L	31,995 (24,234; 43,757)	26,310 (21,282; 30,908)	25,503 (11,303; 32,086)
TGFa, pg/L	7.1 (3.4; 13.4)	3.9 (1.8; 6.5)	2.4 (0.0; 3.7) *
TNFa, pg/L	52.5 (40.8; 71.7)	29.8 (19.8; 38.6) *	22.6 (16.6; 29.1) *
TNFb, pg/L	5.7 (4.4; 8.2)	4.4 (0.0; 10.2)	3.6 (0.0; 6.9)
VEGF-A, pg/L	169.0 (71.3; 380.1)	50.2 (26.9; 116.7)	48.5 (24.3; 92.9) *

* *p* < 0.01 compared with acute COVID-19. ^ *p* < 0.01 compared between convalescents.

**Table 2:** Comorbidities diversity in patient’s group.

Associated Comorbidities	Acute COVID-19, N (%)	Convalescent, N (%)
Arterial hypertension	65 (70)	63 (72)
Ischemic heart disease	24 (26)	15 (17)
Obesity (body mass index > 30)	31 (33)	40 (45)
Type 1 diabetes mellitus	1 (1)	0 (0)
Type 2 diabetes mellitus	29 (31)	22 (25)
Chronic kidney disease	6 (6)	8 (9)
Chronic hepatitis	1 (1)	0 (0)
Chronic obstructive pulmonary disease	1 (1)	0 (0)

**Table 3:** Clinical and laboratory data.

Parameters (Reference Values)	Moderate COVID-19 (n = 68)	Severe COVID-19 (n = 25)	3–6 Months (n = 40)	6–12 Months (n = 48)	Healthy Donors (n = 27)
Age, years	59.5 (53; 70)	59 (56; 70)	56 (49; 64)	60.5 (51; 71)	37.0 (32.0; 47.0)
Hemoglobin, g/L	135.5 (126; 142)	132 (125; 143)	144 (135; 153)	143 (135; 153.5)	139.5 (136; 148)
Red blood cells × 10^12^/L	4.74 (4.48; 5.045)	4.61 (4.48; 4.84)	4.9 (4.68; 5.27)	4.835 (4.645; 5.185)	5.00 (4.70; 5.13)
Hematocrit, %	40.35 (37.1; 41.9)	39.3 (37.1; 41.3)	42.6 (41; 44.9)	41.5 (40.35; 44.7)	43.2 (41.5; 46.0)
Platelets × 10⁹/L	188 (140; 250.5)	201 (157; 290)	213 (193; 245)	236.5 (188.5; 277)	208 (194; 235)
Leukocytes × 10⁹/L	5.31 (4.05; 7.95)	8.6 (6.6; 10)	6.62 (5.26; 7.62)	6.075 (5.27; 7.705)	5.8 (5.4; 7.7)
Lymphocytes × 10⁹/L	1.175 (0.905; 1.55)	1.03 (0.72; 1.24)	1.99 (1.53; 2.45)	1.585 (1.31; 1.91)	1.95 (1.78; 2.21)
Monocytes × 10⁹/L	0.56 (0.305; 0.715)	0.37 (0.17; 0.69)	0.47 (0.41; 0.67)	0.61 (0.48; 0.72)	0.58 (0.39; 0.71)
Neutrophils × 10⁹/L	3.45 (2.535; 5.25)	7.56 (4.71; 8.18)	3.54 (2.93; 4.62)	3.705 (3.125; 4.92)	2.96 (2.78; 4.31)
Eosinophils × 10⁹/L	0.01 (0; 0.04)	0 (0; 0.03)	0.13 (0.08; 0.21)	0.17 (0.11; 0.35)	0.11 (0.08; 0.23)
Basophils × 10⁹/L	0.01 (0.005; 0.01)	0.01 (0.01; 0.02)	0.01 (0.01; 0.01)	0.01 (0.005; 0.01)	0.08 (0.06; 1.00)
CRP, mg/L (0–5.0)	29.81 (12.03; 51.59)	127.13 (32.41; 216.3)			
D-dimer, mg/μL (0–5.0)	0.24 (0.14; 0.49)	0.445 (0.29; 0.765)			
Ferritin, ng/mL (30–400)	424 (202.15; 784.4)	609.7 (330.3; 1002)			
Procalcitonin, ng/mL (0–0.5)	0.070 (0.047; 0.100)	0.098 (0.064; 0.22)			
ALT, U/L (0–41)	26.4 (15.5; 42.8)	22.3 (17.0; 40.1)			
AST, U/L (0–40)	28.1 (21.9; 39.8)	27.7 (22.7; 41.9)			
LDH, U/L (135–225)	320 (251.5; 456)	306 (251; 422)			
Computed tomography, % of lung involvement	25 (15; 40)	50 (35; 55)			

## Data Availability

Data is contained within the article.

## Abbreviations

SARS-CoV-2, severe acute respiratory syndrome coronavirus 2; COVID-19, Coronavirus disease 2019; ARDS, acute respiratory distress syndrome, Tregs, T regulatory cells; CM, central memory; EM, effector memory; TEMRA, terminally differentiated CD45RA^+^ effector cells; CCL2, C-C motif chemokine ligand 2; CCR, C-C motif chemokine receptor; CXCR, C-X-C motif chemokine receptor; CD, cluster of differentiation; CTLA-4, cytotoxic T-lymphocyte associated protein 4; FOXP3, forkhead box P3; G-CSF, granulocyte colony-stimulating factor; GM-CSF, granulocyte macrophage colony-stimulating factor; M-CSF, macrophage colony-stimulating factor; TGF-β, transforming growth factor-beta; Ki-67, Antigen Kiel 67; GITR, Glucocorticoid-Induced TNFR-Related; IFN, interferon; IL, interleukin; ICOS, Inducible T-cell COStimulator; KLRG1, killer cell lectin-like receptor G1; PD1, programmed cell death protein 1; MDSC, myeloid-derived suppressor cell; CTL, Cytotoxic T lymphocytes; PCR, polymerase chain reaction; DNA, Deoxyribonucleic acid; CRP, C-reactive protein; LDH, lactate dehydrogenase; ATP, adenosine triphosphate; ADP, adenosine diphosphate, AMP, adenosine monophosphate; ROS, reactive oxygen species; Th1, T helper 1; TNF-α, tumor necrosis factor alpha.
